# To: Admission factors associated with intensive care unit readmission in critically ill oncohematological patients: a retrospective cohort study

**DOI:** 10.5935/0103-507X.20160061

**Published:** 2016

**Authors:** Müge Aydoğdu, Antonio M. Esquinas

**Affiliations:** Department of Pulmonary Diseases, Faculty of Medicine, Gazi University - Ankara, Turkey.; Intensive Care Unit, Hospital Morales Meseguer - Murcia, Spain.

**To the editor**

We read the manuscript by Rodrigues et al. in the latest issue of Revista Brasileira de Terapia Intensiva Journal with great interest.^(1)^ In this single-center, retrospective, observational cohort study, Rodrigues et al. aimed to identify risk factors associated with later readmission to the intensive care unit (ICU) among critically ill oncohematological patients by evaluating their first ICU admissions. They identified male sex, emergency surgery as the admission reason, longer length of hospital stay before ICU transfer and mechanical ventilation (MV) as independent risk factors for ICU readmissions. The hypothesis of this study was attractive because it evaluated a specific group of patients, "oncohematological patients," who were followed in multiple critical care units and had different characteristics from the typical medical and surgical ICU populations. However, by evaluating only the admission factors of their first ICU stay, Rodrigues et al. limited their results to a narrow window. They disregarded evaluating other more important risk factors, some of which were mentioned in the manuscript's discussion section.

When evaluating readmission to ICU for a specific patient group, Gajic et al.^(2)^ categorized the possible risk factors into 3 groups. These were (1) first ICU admission characteristics, (2) physiological characteristics, laboratory abnormalities and severity of illness at the time of ICU discharge and (3) functional status and need for nursing interventions at the time of ICU discharge.

According to this categorization, Rodrigues et al.^(1)^ should also have presented the first ICU admission characteristics of their patient group. They identified the length of hospital stay before ICU transfer as an independent predictor of readmission but did not take into consideration the length of ICU stay or the length of MV during patients' first ICU admission. They also did not mention the type of MV applied, whether invasive MV (IMV) or noninvasive MV. Another risk factor that was excluded was ventilator-associated pneumonia development during the first ICU stay. Other relevant risk factors in oncohematological patients include immune suppression status and infections associated with this immune suppression, such as cytomegalovirus infections, pneumocystis carinii pneumonia, Aspergillus infections or invasive candidiasis. These infections are challenging to eradicate, and if not properly eradicated, can lead to readmissions in this group of patients.

Second of all, physiological characteristics, laboratory abnormalities and severity of illness at the time of ICU discharge were not evaluated as risk factors of readmission by Rodrigues et al.^(1)^ In assessing readmissions, it is important to consider characteristics of the patients' discharge status, including the hemodynamic status, laboratory parameters for arterial blood gas analysis, discharge Simplified Acute Physiology Score (SAPS 2) or Acute Physiology and Chronic Health disease Classification System II (APACHE II) score, discharge organ dysfunction status (liver and renal functional tests and neurological status) and discharge infection and immune suppression status.^(2-5)^ The Stability and Workload Index for Transfer (SWIFT) score, as Rodrigues et al. also mentioned in their discussion, would be more suitable for evaluating readmissions^(2,3)^ in this study. Their reason for excluding this score was that routine arterial blood gas analysis was not routinely performed at discharge. We believe, however, that in most ICUs, patients usually undergo evaluation of final arterial blood gas analysis prior to discharge. Therefore, the study authors could have taken into consideration the results of the last arterial blood gas analysis before discharge. In addition to the SWIFT score, the Modified Early Warning Score (MEWS), a modified SWIFT score that includes renal function, and the SOFA score (which evaluates organ dysfunction) at discharge could be appropriately included in this study.^(3)^

Although Rodrigues et al. mentioned in their discussion that oncohematological patients are more susceptible to post-ICU complications that require readmission, such as treatment-related immune suppression, cancer-associated malnutrition, invasive procedures, repeated surgeries, and increased thrombotic tendency, they did not include these important parameters into univariate and multivariate analyses.^(1)^ We believe that these parameters would reflect the readmission risk factors of oncohematological patients more effectively than the rest of the parameters that were included in this study.

Third, it is important to assess the functional status and need for nursing interventions at the time of ICU discharge. The discharge frailty status (bedridden or mobilized); the discharge facility (ward, intermediate ICU or a palliative care unit); and the discharge status of the patients (presence of tracheotomy, invasive or noninvasive home mechanical ventilator or oxygen concentrator use) should also be evaluated as risk factors of ICU readmission.

In addition to first admission characteristics, all possible risk factors for ICU readmission of oncohematological patients should be investigated with further comprehensive studies.

Müge Aydoğdu

Department of Pulmonary Diseases, Faculty of Medicine, Gazi University - Ankara, Turkey.

Antonio M. Esquinas

Intensive Care Unit, Hospital Morales Meseguer - Murcia, Spain.
